# Reducing incidence of cervical cancer: knowledge and attitudes of caregivers in Nigerian city to human papilloma virus vaccination

**DOI:** 10.1186/s13027-018-0202-9

**Published:** 2018-08-17

**Authors:** Adaobi I. Bisi-Onyemaechi, Ugo N. Chikani, Obinna Nduagubam

**Affiliations:** 1 College of Medicine, University of Nigeria Ituku-Ozalla, Enugu, Nigeria; 2 College of Medicine, Enugu State Teaching Hospital Parklane, Enugu, Nigeria

**Keywords:** Awareness, Human papilloma virus, Cervical cancer, Vaccines, Nigeria

## Abstract

**Background:**

Despite the high prevalences of Human Papilloma Virus (HPV) infections and cervical cancer in Nigeria, utilization of the HPV vaccine as a highly effective preventive measure remains low. The aim of this study was to find out the awareness and attitudes of caregivers to HPV infections and the factors that determine acceptance of an HPV vaccine for their pre-adolescent girls.

**Methods:**

This was a cross-sectional descriptive study of 508 caregivers of female children in Enugu Nigeria. A semi-structured questionnaire was used to collect information on knowledge of HPV, cervical cancer as well HPV vaccine and its acceptance for pre-adolescent female children. The data was analysed using descriptive statistics.

**Results:**

Five hundred and eight (508) caregivers of female children were interviewed. Less than half, 221,(43.5%) of them knew about HPV, among these, 163 knew how HPV is transmitted. Only 12 (2.4%) of the caregivers know that an HPV infection is a major risk factor for cervical cancer. Among the 221 participants who knew the meaning of HPV, 132 (59.7%) were aware of an HPV vaccine. Only 26 (19.7%) of those aware of a vaccine agreed it can effectively prevent cervical cancer. Lack of awareness about the vaccine and accessibility were the major reasons given by parents on why the vaccine has not been received by their female children.

**Conclusion:**

Despite high levels of education, awareness of HPV, HPV vaccine and the risks for cervical cancer remains low among caregivers in Enugu, south-east, Nigeria. Awareness and accessibility were the major determinants of HPV vaccine uptake among the caregivers. There is a need for massive and sustained awareness creation to increase HPV vaccination uptake in Nigeria.

## Background

Human Papilloma Virus (HPV) is the most common sexually transmitted virus and it is estimated that about 75% of sexually active women and men will acquire a genital HPV infection at some time [1]. HPV is known to affect adult and children alike and over 100 types of the HPV has been identified over the past few decades [2].

There has been an increasing interest in HPV because of their relationship with tumours particularly cervical. Epidemiological, molecular and clinical evidence has shown that cervical cancer is caused by HPV. About 13genotypes are closely linked with cervical cancer especially genotypes 16 and 18 [3–11]. High risk Infections is usually persistent and should be the target of vaccination strategies [12].

Globally, cervical cancer is a major public health problem. Over 560,000 new cases and about 275,000 deaths are recorded each year, with more than 80% occurring in developing countries [13, 14]. It is the most common gynaecological cancer among women in sub-Saharan Africa [15]. It is estimated that 70,722 new cases of invasive cervical cancer occur annually in sub-Saharan Africa [16]. According to GLOBOCAN 2008, about14,089 new cervical cancer cases are diagnosed annually in Nigeria.

Cervical cancer is the second most common cancer in Nigeria and second to breast cancer among its female population [17–21]. In 2007, it was reported that 36.59 million women aged more than 15 years in Nigeria are at risk of developing cervical cancer. There are 9922 cases diagnosed annually with 8030 deaths. HPV prevalence is 24.8%. Incidence of cervical cancer in Nigeria is 250/100,000 women [22]. One of the preventive measures is the vaccination of pre-adolescents against oncogenic HPV. The vaccines are approved for administration to persons aged 9–26 years [9, 23, 24]. The target is to commence the vaccine among young children before they become sexually active. The cervical cancer control plan in Nigeria recommends visual inspection with acetic acid (VIA) or with Lugol’s iodine, for screening, (secondary prevention strategy) of sexually-exposed women, and for primary prevention, HPV vaccination for girls aged 9 to 15 years. HPV vaccination has not been introduced in the vaccination schedule in Nigeria and is only available to people on personal arrangements. Programs are set up to vaccinate girls on adhoc, irregular and usually private basis.

A large number of European countries as well as United States, Australia and New Zealand have recommended including an HPV vaccine in the school vaccination program for young adolescent girls, often coupled with a catch-up program for older teenage girls [25, 26]. The vaccine has been found 70–100% effective in preventing cervical cancers [27–30]. The vaccines were licensed and introduced in Nigeria in 2009, uptake has ranged between 0 and 49% being utilized by only a few privileged population [30–32].

The knowledge of HPV infections and HPV vaccines, among the Nigeria population, are inadequate, and the cost of HPV vaccination per person is beyond what the average Nigerian can afford [33–36].

Awareness and knowledge of the infections and the vaccines would stimulate demand and uptake of the vaccines. Increasing demand may drive the introduction of the vaccine into the national immunization schedule thereby making the vaccine more affordable and accessible.

The aim of this study was to find out the knowledge and attitudes of parents to and the factors that determine the demand of HPV vaccine for their adolescent girls. This study was focused on the usefulness of HPV Vaccine as a preventive measure for the reduction of the incidence of cervical cancer hence the focus only on adolescent girls.

## Methods

This was a cross-sectional descriptive study of 508 parents/caregivers of young children from primary schools in the three Local government councils of Enugu metropolis- Enugu-South, Enugu- North and Enugu-East. Fifteen primary schools were (five from each local council) were randomly selected for the study. The researchers attended the Parents- Teachers forum of these schools. During the meeting, the researchers introduced themselves and the purpose of their research. They were then granted permission to administer the questionnaire to consenting parents of female children. Every eligible caregiver of a female child less than 18 years was recruited into the study. The data was collected consecutively from school to school. Data was collected between the months of January and July 2017. Data collection tool was semi-structured questionnaire reviewed by a panel of experts for validity. The questionnaire was either self-administered or interviewer-administered for study participants who were not literate. Items on the questionnaire include; socio-demographic variables; knowledge of cervical cancer; knowledge of HPV infections and HPV vaccines, their attitudes towards the effectiveness of the vaccines and reason (s) for uptake or otherwise of these vaccines for their adolescent girls. The data was analysed using descriptive statistics.

## Results

A total of 508 caregivers were interviewed out of which 476 (93.7%) were below the age of 50 years. 385 (75.8%) were females and 123 (24.2%) males. Out of the 508 caregivers, 218 (42.9%) had more than one female child. The mean age of the children of the respondents was 7.3 years (SD + − 5.3) Three hundred and forty-seven (68.3%) had tertiary level of education while only 30 (5.9%) had no formal education. Three hundred and thirty (65%) caregivers were married (Table 1).

**Table 1:** Socio-demographic information of study participants

	Frequency	Percent
Age group		
< =25	116	22.8
26–30	86	16.9
31–35	94	18.5
36–40	87	17.1
41–45	62	12.2
46–50	31	6.1
> 50	32	6.3
Sex		
Male	123	24.2
Female	385	75.8
No of female children		
1	290	57.1
2	22	4.3
3	50	9.8
4	67	13.2
> 4	79	15.6
Level of education		
None	30	5.9
Primary	29	5.7
Secondary	102	20.1
Tertiary	347	68.3
Marital Status		
Single	154	30.3
Married	330	65.0
Divorced	7	1.4
Separated	17	3.3

A total of 221 (43.5%) parents knew what HPV meant, among these, 163(74%) knew how it is transmitted (Tables 2 and 3).

**Table 2:** Knowledge about HPV

What is HPV	Frequency	Percent
Don’t know	200	39.4
Human papilloma virus	221	43.5
It is a very bad disease	1	0.2
It is a virus and very deadly disease	1	0.2
Hepatisis vaccine	44	8.7
Human productivity virus	25	4.9
High power voltage	1	0.2
High productivity vaccine	8	1.6
Human power	1	0.2
A vaccine	1	0.2
Human positive virus	2	0.4
Hypertension	2	0.4
Human production value	1	0.2

**Table 3:** Knowledge about the risk factors for HPV infection

What are the risk factors for HPV infection?	Frequency	Percent
Don’t know	89	40.3
Unprotected sex and multiple partners	20	9.0
Frequent cervical contact	1	0.5
Cancer and genital warts	15	6.8
Death	22	10.0
Promiscuity	58	26.2
Adolescence	2	0.9
Use of condom	1	0.5
Infection	2	0.9
In the womb	1	0.5
Early sex	1	0.5
Child birth	1	0.5
Stroke	1	0.5
Bleeding	1	0.5
Sickness	2	0.9
Vaginal discomfort	4	1.9
Total	221	100.0

Overall, only 21 (9.5%) of the parents who were aware of HPV knew the risk factors for genital HPV infections while a total of 66 (30%) of these knew that the risk of developing invasive cervical cancer is the major “sequale” following HPV infection (Tables 3 and 4).

**Table 4:** Knowledge of the sequale of genital HPV infections

What is the hazard for HPV infection?	Frequency	Percent
Don’t know	96	43.4
Risk of having cervical cancer	66	29.9
Death	51	23.1
Multiple sex partner	1	0.5
Infection	2	0.9
Yellow fever in fetus	1	0.5
Injury in the cervix	1	0.5
Bleeding	1	0.5
Chemical radiation	1	0.5
Genital warts	1	0.5
Total	221	100.0

Although 361(71%) of the respondents have heard about cancer of the cervix, 65% of them did not know what it means. Only 12 out of the 221(5.4%) participants that know about HPV are aware that it is risk factor for developing cervical cancer. These 12 were part of the 66 who identified that risk of developing cervical cancer as a sequale of genital HPV infections (Table 5).

**Table 5:** Knowledge about the risk factor for cervical cancer

What are the risk factors for cervical cancer?	Frequency *n* = 508	Percent
Don’t know	265	52.2
Death	98	19.3
Sexual transmitted disease/unprotected sex	7	1.4
Early sex and multiple sex partners	4	.8
Multiple sexual partners	5	1.0
Sexual promiscuity	1	.2
Infection	62	12.2
Neck injury	1	.2
Cancer of the cervix	3	.6
Injury in the cervix	20	3.9
Pain in the cervix/womb	17	3.3
Affection	1	.2
Family planning inserts	2	.4
Vaginal Bleeding	3	.6
HPV infection	12	2.4
Instrumentation in female private part	2	.4
Sickness	1	.2
Low fertility	2	.4
Heredity	1	.2
Damage of the uterine wall	1	.2

Among the 221 participants who knew the meaning of HPV, 132 (59.7%) were aware of HPV vaccine. Furthermore, of the 132 participants who are aware of the HPV vaccine; only 26 (19.7%) agree that HPV vaccine can effectively prevent cervical cancer; 59(44.7%) didn’t think so while 47(35.6%) had no idea.

Among those aware of the vaccine, 72 (54.5%) do not know what ages the HPV vaccine should be administered and only 6(2.75%) of the parents had given HPV vaccine to their eligible female children.

The major reasons given by parents/caregivers for not giving HPV vaccine to their eligible children were that they were not aware of the vaccine (74.1%) and inability to access the vaccine among those aware of it. (72.5%) (Table 6).

**Table 6:** Reason (s) for not giving HPV vaccine by those aware of the vaccine

REASON	Frequency *n* = 132	Percent
I don’t know how to access the vaccine	123	93.2
My daughter is too young to have risk of cervical cancer	94	71.2
I am worried about the safety of the vaccine	47	35.6
I am not sure of the effectiveness of the vaccine	26	19.7
The decision would be made by the child herself	108	81.8
The vaccine is expensive	25	18.9

## Discussion

It was observed that many the study participants had formal education. The finding that 68.3% of the respondents had tertiary level of education signifies a high literacy level. However, despite this, awareness of cancer of cervix amongst them was low. This finding is similar to other studies in Nigeria, among women with similar social characteristics (age and educational attainment) where only a small proportion of respondents knew about the disease despite high levels of literacy [11, 36]. However, in contrast, a similar study Ibadan that reported that awareness of cancer of cervix was up to 67% despite lower literacy level among the study participants [30, 35]. Another study in Lagos Nigeria reported an awareness level of up to 99% among nurses which is not unexpected as they were health workers with more access to health information [36].

Awareness about HPV infection was also low in this study; very few caregivers knew the risk factors for HPV infection as well as the relationship between HPV infection and cervical cancer. This may be because it is not part of routine health talks at ante-natal or immunization sessions. These are the major places where most mothers get health information in this environment with limited access to internet and electricity. Similar finding of low awareness about HPV were also found in Lagos and Malaysia where only less than half of the mothers with similar educational attainment were aware of HPV disease [34]. However, in Ibadan Nigeria, awareness of HPV disease was found to be high [30]. This low level of awareness among the educated implies that a lot of information, communication and education strategies have tobe engaged to enlighten the public on a prevalent disease like cervical cancer. Health promotion strategies to educate the public about prevention of STIs of public health significance can be effective in preventing genital HPV infection and by extension cervical cancer [24].

Primary prevention of cervical cancer can be achieved through prevention and control of genital infection with oncogenic HPV types [24]. Oncogenic genital HPV infections can be prevented by vaccinating young female children with HPV vaccine. In Nigeria, mothers influence decision making particularly for their young female children; hence places with high concentration of mothers like antenatal classes, markets and parent-teachers forum meetings can be engaged to improve HPV awareness.

This study is similar to a number of other studies which have shown that awareness of the vaccine is also very low [11, 34, 35]. Also, more 90% of the respondents who were aware of the HPV vaccine cannot access it. This is worrisome because effective coverage of HPV vaccination requires both caregiver acceptance of the vaccines [5, 9] accessibility and affordability. Uptake rates cannot be improved when basic knowledge is lacking and vaccines inaccessible. This would result in underutilization of this preventive measure as was the case in this study.

As much as 45% of those aware of the vaccine do not agree that HPV vaccine can effectively prevent cervical cancer. Cost of the vaccine, concerns about the effectiveness and safety of the vaccine were reasonsgiven by the caregivers for poor uptake of HPV vaccines. Previous studies [33–36] have highlighted cost concerns as a major issue impeding the uptake of HPV vaccine, this study reveals knowledge gaps about the vaccine and accessibility as the more important factors than cost of the vaccine in our environment. Currently in Nigeria, HPV vaccine is optional, not included in the national immunization schedule and also not subsidized by government. Agida et al. [11] in their study observed that regardless of the current cost of the vaccine in Nigeria, acceptance was high among parents who were aware of the vaccine. Adequate information also needs to be provided to the parents and caregivers to dispel all wrong perceptions about HPV vaccine.

Awareness campaigns in places like ante-natal clinics, parents-teachers forums of schools etc., particularly amongst caregivers of young female children is pivotal to improving uptake of HPV vaccination and reducing incidence of cervical cancer in Nigeria. Governments should subsidize and include the HPV vaccine in National immunization schedules to encourage uptake and subsequently reduce the incidence of cervical cancer.

## Conclusions

Despite high levels of education, the following, awareness of HPV infections and cervical cancer, and their related risk factors and HPV vaccines were low among caregivers of female children in Enugu, south-East, Nigeria.

Similarly history of HPV vaccination among the children of the respondents was low, accessibility and affordability was the most common challenge among those aware of the vaccine. There is a need for massive and sustained awareness creation to step up HPV vaccination in Nigeria.

## Abbreviations

DNA: Deoxyribonucleic Acid
HPV: Human Papilloma Virus
LGA: Local government areas
STI: Sexually transmitted infection
